# Microwave-Assisted Tunneling in Hard-Wall InAs/InP Nanowire Quantum Dots

**DOI:** 10.1038/s41598-019-56053-2

**Published:** 2019-12-20

**Authors:** Samuele Cornia, Francesco Rossella, Valeria Demontis, Valentina Zannier, Fabio Beltram, Lucia Sorba, Marco Affronte, Alberto Ghirri

**Affiliations:** 1 Dipartimento di Scienze Fisiche Informatiche e Matematiche, Università di Modena e Reggio Emilia, via G. Campi 213/A, 41125 Modena, Italy; 2 Istituto Nanoscienze - CNR, via G. Campi 213/A, 41125 Modena, Italy; 3 NEST, Scuola Normale Superiore and Istituto Nanoscienze - CNR, Piazza San Silvestro 12, 56127 Pisa, Italy

**Keywords:** Electronic devices, Nanowires

## Abstract

With downscaling of electronic circuits, components based on semiconductor quantum dots are assuming increasing relevance for future technologies. Their response under external stimuli intrinsically depend on their quantum properties. Here we investigate single-electron tunneling in hard-wall InAs/InP nanowires in the presence of an off-resonant microwave drive. Our heterostructured nanowires include InAs quantum dots (QDs) and exhibit different tunnel-current regimes. In particular, for source-drain bias up to few mV Coulomb diamonds spread with increasing contrast as a function of microwave power and present multiple current polarity reversals. This behavior can be modelled in terms of voltage fluctuations induced by the microwave field and presents features that depend on the interplay of the discrete energy levels that contribute to the tunneling process.

## Introduction

Single (few) electron transistors based on semiconductor quantum dots (QDs) are flexible solid-state components characterised by extensive control of charge, orbital and spin degrees of freedom. Electrons fill the dot in a shell structure in analogy with artificial three dimensional atoms and their wavefunctions depend on the shape and size of the system^1–3^. Orbital properties, in turn, determine the tunneling current and more in general the QD response to external stimuli. Bottom-up grown InAs nanowires (NWs) recently emerged as a reliable platform to produce single^3–6^ and double^7,8^ QDs with strong electron confinement. In the case of high aspect ratio NW QDs, lowest lying states have dominant radial character while an axial component can characterise their excited states^3^. Typically, the separation of their energy levels can be controlled between few tenths to tens of meV during growth^9^, while further tuning of NW QD energy levels can be obtained by (multi) gating^5^. Heterostructured InAs/InP NW QDs are characterised by hard-wall confinement potential and large single-particle energy spacing with Coulomb and Pauli blockade detectable up 50 and 10 K, respectively^5,8^. Thanks to their large spin-orbit coupling, InAs NW QDs have been proposed as spin qubits with electric control of the spin degree of freedom^10,11^.

QDs coupled to microwaves (MW) for spintronics and quantum technologies have been also proposed and developed^12–17^. When the photon energy $\hslash$*ω* matches the level spacing, coherent and/or resonant phenomena may occur and NW QDs may function either as single-atom maser sources^18–20^ or as photon detectors in the MW range^21^. Non-resonant MW excitation may assist tunneling process thus affecting the charge transport characteristics, as observed on other semiconductor quantum well^22^ and QD systems: effects of electromagnetic radiation on Coulomb blockade peaks were reported for electrostatically defined GaAs QDs^23–25^ and single-walled carbon nanotubes^26,27^. The lifting of Coulomb blockade in the presence of MW radiation can be described in terms of photon-assisted tunneling (PAT)^28^ that, in the quantum limit ($\hslash$*ω* > *k*_*B*_*T*), gives rise to inelastic single-electron tunneling^29^. In the high MW power regime, sidebands of the main Coulomb peaks appear and excited states within the QD can contribute to assist the tunneling process^24^. This situation was not explored so far in heterostructured InAs/InP NW QDs despite their significant technological potential.

Here, we first characterise the stability diagram of InAs/InP NW QDs; we then study Coulomb-blockade lifting in the presence of an off-resonant MW drive. We find that MW effects exhibit a power threshold and, under suitable conditions, current polarity reversal can be observed. Our results show that MW assisted tunneling can take place in non-resonant conditions as an effect of the voltage fluctuations of the microwave field and may depend on the presence of excited states of the QD involved in the tunneling process.

## Results

### Device description and characterisation

**Figure 1:**
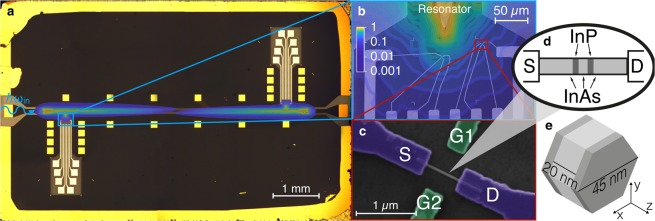
(**a**) Optical microscope image of the whole electrical device with central YBCO/sapphire coplanar resonator. Gold contacts and bonding pads are used for connecting dc lines and the coplanar resonator to the external cables. (**b**) Scanning Electron Microscope (SEM) close-up showing the antenna tip and the leads of two NW QD devices. In panels (a) and (b) the finite-element simulation of the distribution of the electric component of the fundamental mode of the resonator is superimposed with false colours. The colour scale is normalized to the maximum value. (**c**) False colour SEM image of the NW QD device investigated in this work, where source (S), drain (D) and gate (G1, G2) leads are indicated. (**d**) Schematic diagram of the InAs/InP NW QD. (**e**) Sketch of the NW QD showing InAs (light grey) and InP (dark grey) sections.

In order to optimise the coupling with microwaves, NW QD devices were fabricated in proximity of a half-wavelength superconducting coplanar resonator. The resonator was realised out of a 8 × 5 mm YBCO/sapphire film and presents a 6 mm-long central conductor, having width of 30 *μ*m and distance of 55 *μ*m from the lateral ground planes. The fundamental mode has resonance frequency *ω*_0_/2*π* = 9.815 GHz (Supplementary Material). QD devices are fabricated in correspondence of the electric antinodes of the fundamental mode of the resonator, where two tips connected to the central strip are designed to work as “antenna”, in order thus to extend and enhance the microwave field in proximity of the NW QD device (Fig. 1(a,b)). Up to four QD devices can be individually tested for each chip. In this work we report results obtained on one of our NW QD devices, positioned at 65 *μ*m from the antenna tip (Fig. 1).

**Figure 2:**
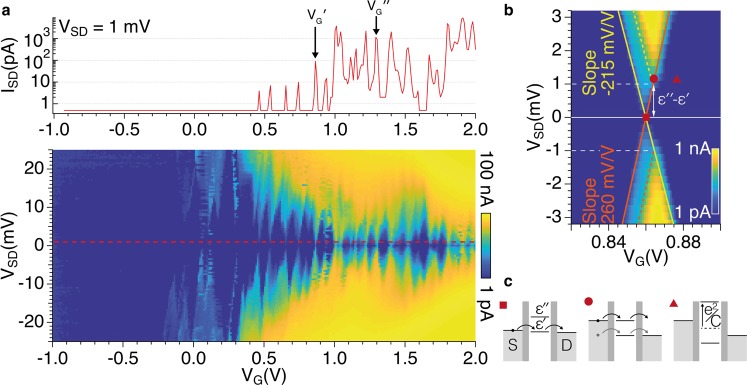
(**a**) Stability diagram (bottom panel) and crosscut at *V*_*SD*_ = 1 mV (top panel) of the log(|*I*_*SD*_|) current measured at *T* = 2 K. (**b**) Blow-up of the current map measured in the proximity of the peak at ${V^{\prime} }_{G}$ = 0.86 V. The colour scale is logarithmic. (**c**) Schematic diagrams showing the configuration of the electrochemical potentials of ground (*ε*′) and first excited (*ε*″) states in correspondence of the symbols in panel (b).

In order to characterise the QD we first measured the dc current (*I*_*SD*_) as a function of bias voltage (*V*_*SD*_) and gate voltage (*V*_*G*_). The same voltage was applied simultaneously to both side gates (*V*_*G*1_ = *V*_*G*2_ = *V*_*G*_). The stability diagram of the NW QD measured at the temperature *T* = 2 K presents a typical Coulomb-diamond structure (Fig. 2(a)). The addition energy of the *N*-th electron in the QD can be estimated in the framework of the constant interaction model^30^:

1 $${\mu }_{N}={E}_{k}+\frac{N{e}^{2}}{C}-|e|\alpha {V}_{G}+c,$$

where *E*_*k*_ is the energy of the first available QD level, *C* is the capacitance of the QD and *c* is a constant. The lever arm *α* is a geometry-dependent parameter that accounts for the effect of gate voltage on QD levels, which can be obtained from the diamond boundary slopes *m*_1_ and *m*_2_ as 1/*α* = 1/*m*_1_ − 1/*m*_2_. Data in Fig. 2(a) yield average values *α* = 120 ± 20 mV/V and a charging energy *e*^2^/*C* = 9 ± 2 meV. The split of the energy levels in our QD well matches values obtained for hard-wall InAs/InP NW QDs (*ca*. 10 eV) having similar geometry and stronger axial confinement along the growth direction of the NW^5,9^.

The plot of *I*_*SD*_ at constant voltage bias *V*_*SD*_ = 1 mV is shown in the top panel of Fig. 2(a). As a function of the gate voltage, full depletion and progressive occupation of the QD states can be observed. Although collected data do not allow for a direct correspondence of current peaks with electron levels with spectroscopic precision, we expect that electrons are added to fill in the shell structure of the QD, which is determined by the specific characteristics of the electron orbitals^1–3^.

Data plotted in log-scale in Fig. 2(a) show a marked increase of the peak current from ~10 pA to ~1 nA beyond a threshold voltage of 1 V. These abrupt change reflects the different tunneling rates (Γ) that can be estimated by considering the Fermi-Dirac occupation of the source and drain leads at *T* = 2 K (Supplementary Material). By fitting the *I*_*SD*_(*V*_*SD*_) peaks, we obtain Γ′ = 1 GHz for ${V^{\prime} }_{G}$ = 0.86 V, whilst for *V*_*G*_ > 1 V we estimate $\Gamma ^{\prime\prime}$ ≈ 10 GHz. These numbers are consistent with what previously observed in similar QD devices^5^.

The stability diagram taken as function of the gate voltage for *V*_*G*_ ~ ${V^{\prime} }_{G}$ is shown in Fig. 2(b). The diamond exhibits two different slopes (*m*_1_ = −215 mV/V and *m*_2_ = 260 mV/V) that correspond to slightly asymmetric lever arms for source and drain leads. Figure 2(b) shows that *I*_*SD*_ ∝ Γ′*V*_*SD*_ for voltage gate *V*_*G*_ = ${V^{\prime} }_{G}$ and bias |*V*_*SD*_| < 1 mV, whilst a steeper dependence *I*_*SD*_ ∝ $\Gamma ^{\prime\prime}$*V*_*SD*_ is observed for |*V*_*SD*_| > 1 mV. This behavior suggests that the Coulomb peak is related to tunneling through two charge states with the presence of an excited level at energy Δ*E*_*k*_ = *ε*″ − *ε*′ ≈ 1 meV.

The electron tunneling through the QD can be depicted as in Fig. 2(c), which shows the configuration of the electrochemical potentials *μ*(*ε*′) and *μ*(*ε*″) as a function of the gate voltage around ${V^{\prime} }_{G}$. For *V*_*G*_ = ${V^{\prime} }_{G}$ and *V*_*SD*_ = 1 mV (square symbol in Fig. 2(b,c)), the tunneling involves only the energy level *ε*′, thus the low tunneling rate Γ′ gives rise to a low tunneling current. By expanding the bias window (|*V*_*SD*_| > 1 mV) (circle symbol), the transmission channel through *ε*″ becomes accessible and contributes to the electron transport. A sudden increase of the tunneling current is observed as a consequence of the larger tunneling rate $\Gamma ^{\prime\prime}$. When |*V*_*G*_ − ${V^{\prime} }_{G}$| is sufficiently large (triangle symbol), the level *ε*′ is permanently filled and the addition of an extra electron costs the charging energy *e*^2^/*C*. In this case, *ε*″ is no longer accessible and the current flow is blocked.

### Microwave-Assisted transport

**Figure 3:**
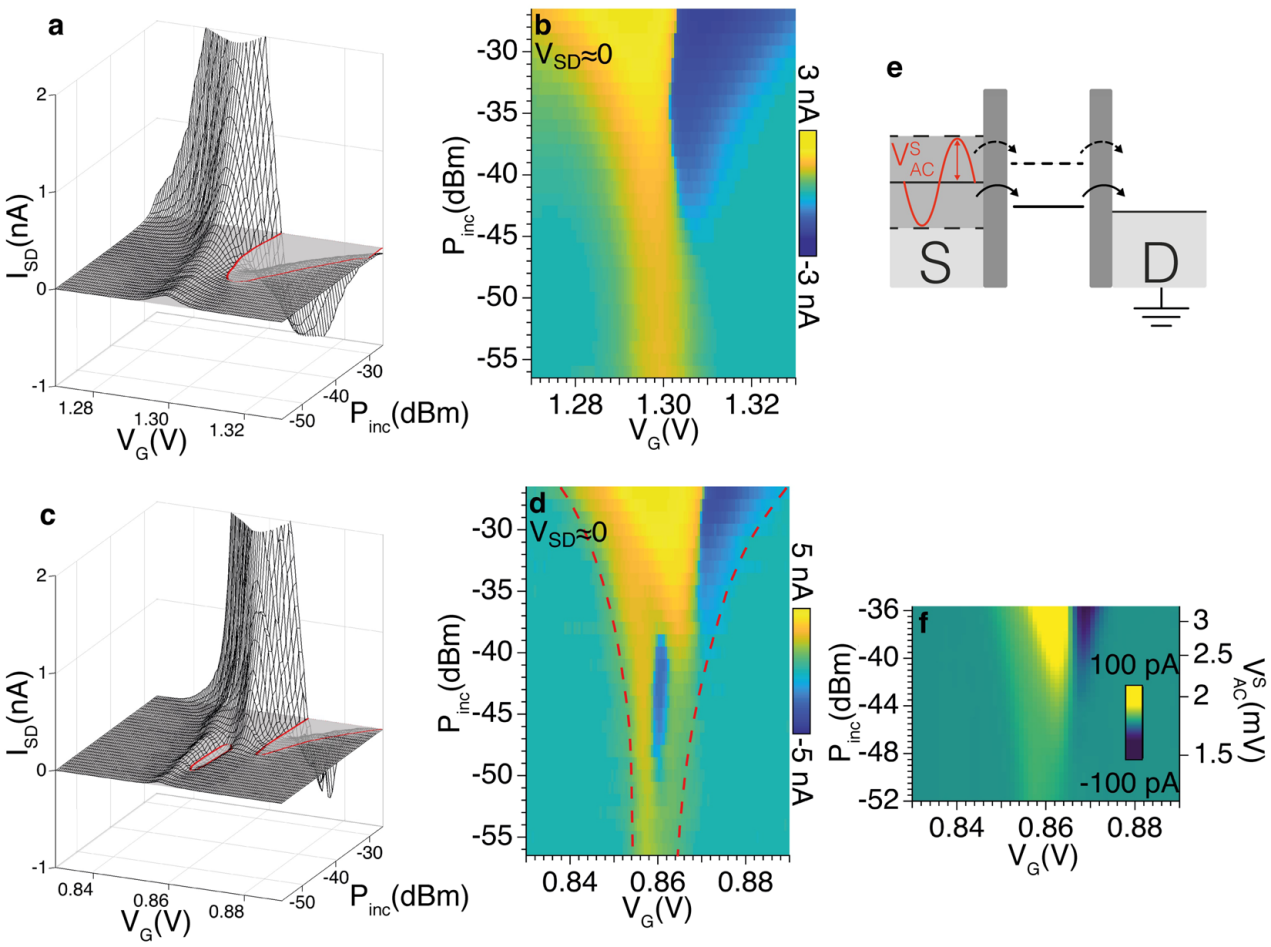
Evolution of the *I*_*SD*_(*V*_*G*_) characteristics in the presence of a microwave drive of frequency *ω*_0_ and increasing power *P*_*inc*_. Three dimensional plots and maps are measured for gate voltage around ${V^{\prime\prime} }_{G}=1.30$ V (**a**,**b**) and ${V^{\prime} }_{G}$ = 0.86 V (**c**,**d**) at the temperature *T* = 2 K. Solid lines indicate the contour of regions with negative *I*_*SD*_. The dashed line in panel (d) display the zero current points used to extract the peak width Δ*V*_*G*_. (**e**) Schematic energy diagrams showing the MW-assisted tunneling through the dot levels *ε*′ and *ε*″. (**f**) *I*_*SD*_(*V*_*G*_) characteristics calculated for *V*_*SD*_ ≃ 0 by averaging the measured current over increasing voltages ${V}_{AC}^{S}$.

In order to study the effect of the microwaves on the transport properties of InAs/InP NW QDs, current measurements were performed in the presence of a monochromatic wave (*ω*_0_/2*π* = 9.815 GHz). In the following we shall focus on two Coulomb peaks at ${V^{\prime} }_{G}$ = 0.86 V and ${V^{\prime\prime} }_{G}=1.30$ V that are representative of the two electron transport regimes as previously described. We initially focused on the Coulomb peak at ${V^{\prime\prime} }_{G}=1.30$ V to investigate the *I*_*SD*_(*V*_*G*_) characteristics by varying the microwave power (Fig. 3(a,b)). As a general observation, the presence of an intense MW drive leads to amplification of the tunnel current and, as expected, this is particularly evident close to *V*_*SD*_ = 0. No effect on electron transport is detected for *P*_*inc*_ < −55 dBm, while MW impact increased more than linearly with increasing MW power. For *P*_*inc*_ > −45 dBm a polarity reversal of *I*_*SD*_ and the broadening of the Coulomb peak are visible. In particular, a negative current peak appears for *V*_*G*_ > 1.30 V even with *V*_*SD*_ > 0. Such trends are found for all Coulomb-blockade peaks that were investigated.

**Figure 4:**
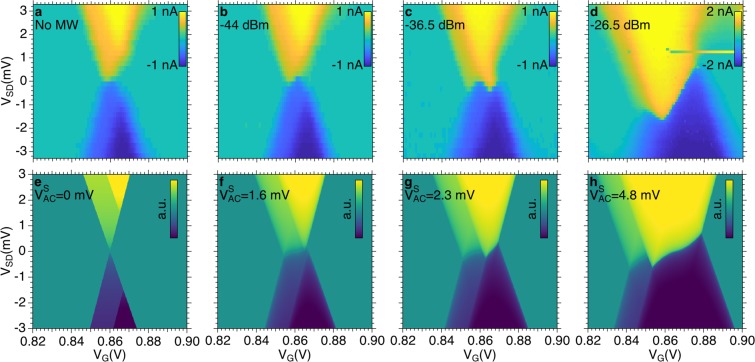
(**a**–**d**) Charge stability diagrams measured around the Coulomb peak at gate voltage ${V^{\prime} }_{G}$ = 0.86 V. (**e**–**h**) Calculated evolution of the stability diagrams in presence of different voltages ${V}_{AC}^{S}$ as reported in the text.

In the case of the Coulomb peak at ${V^{\prime} }_{G}$ = 0.86 V (Fig. 3(c,d)), an additional dip with *I*_*SD*_ < 0 (*i*.*e*. current polarity reversal) is present in the intermediate power range (−50 < *P*_*inc*_ < −38 dBm) and steps appear in the *I*_*SD*_ characteristics for *P*_*inc*_ > −38 dBm. In order to further investigate this point, we mapped *I*_*SD*_ as a function of *V*_*SD*_ and *V*_*G*_ under constant MW power. Figure 4(a–c) shows the evaluation of the Coulomb diamond with increasing *P*_*inc*_. For *P*_*inc*_ = −36.5 dBm two current peaks are visible in panel (c). This effect is enhanced for high power (panel (d)), where the two peaks broaden and merge. The additional dip in Fig. 3(c,d) for −50 dBm < *P*_*inc*_ < −38 dBm can thus be related to the evolution of the diamond shape as a function of the MW power, which gives rise to multiple reversal of the current in the *I*_*SD*_(*V*_*G*_) characteristics for *V*_*SD*_ ≈ 0.

In hard-wall InAs/InP NW QDs Coulomb blockade persists up to *T* ≈ 50 K^5^, thus the effect of the MW field on the transport characteristics can be tested at intermediate cryogenic temperature. At 8 K the Coulomb peaks are much broader, yet the reversal of *I*_*SD*_ is still visible for large power levels (See Supplementary Material). The comparison between 2 and 8 K data shows that the effect of the temperature on the *I*_*SD*_(*V*_*G*_) characteristics is qualitatively different from that of the microwave drive, ruling out the possibility that the observed behavior is simply due to heating.

## Discussion

In NW QDs used in our experiments, the separation between the energy levels of the QD is orders of magnitude larger than the energy of the fundamental mode of the resonator ($\hslash$*ω*_0_ ≈ 40 *μ*eV). This implies that the observed effects (broadening of the Coulomb peak and reversal of the tunneling current) are not related to resonant transitions between single-particle energy levels. On the other hand, the effects of MW are evident for finite intensity of applied electromagnetic radiation: in the many-photon regime and for *k*_*B*_*T* > $\hslash$*ω*_0_, the microwave mode can be treated as a classical electromagnetic field that induces a broadening of the electrodes energy levels^23,24,26,27^. More specifically, the coupling with the microwave field induces an oscillating voltage of amplitude ${V}_{AC}^{S}$ on the source lead. At *V*_*SD*_ = 0, and for a given *V*_*G*_, the current flows when ${V}_{AC}^{S}$ matches the equivalent value of |*V*_*SD*_| that would lift the Coulomb blockade. Therefore the width of the peak results Δ*V*_*G*_ = 2${V}_{AC}^{S}$/min(|*m*_1_|, |*m*_2_|), where *m*_1_ and *m*_2_ are the slopes of the diamond edges. For a given power *P*_*inc*_, ${V}_{AC}^{S}$ can be estimated from the broadening of the Coulomb peak in Fig. 3(d). We calculated the *I*_*SD*_(*V*_*G*_) characteristics by following the method suggested in  references ^26,27^ which consists in calculating, for each *V*_*G*_ value, the average of the current over the ±${V}_{AC}^{S}$ interval. Curves obtained from the experimental data in Fig. 2(b) are displayed in Fig. 3(f). It is worth noting that the main trends of the experimental *I*_*SD*_(*V*_*G*_) data shown in Fig. 3(a–d), in particular current amplification, peak broadening and reversal of current polarity for increasing voltage ${V}_{AC}^{S}$, are reproduced by this simple average calculation. Within the framework of this classical model, the reversal of current polarity emerges as a result of the asymmetry of the Coulomb diamonds (*i.e. m*_1_ ≠ *m*_2_).

More careful inspection of experimental spectra reveals however a more complex behaviour such as a multiple reversal of current polarity, as shown in Fig. 3(c,d) and in the maps in Fig. 4(a–d) and this demands more details. Here it is worth pointing out that multiple reversal of current polarity was observed in coincidence of the Coulomb peak at ${V^{\prime} }_{G}$ (Fig. 2(b)), for which the presence of an additional excited states is evident in the measured stability diagram. Kouwenhoven *et al*.^24^ investigated the process of photon-assisted tunneling with spectroscopic resolution and suggested a model in which excited QD states also contribute to the tunneling. The phenomenological description of stability diagram we previously discussed allows us to extract the essential parameters to describe the effects of classical microwave field also in the presence of excited states in the QD. We firstly reproduce the main features of the DC stability diagram by means of the experimental parameters *m*_1_, *m*_2_, *ε*″ − *ε*′, Γ′ and $\Gamma ^{\prime\prime}$ and we assume *I*_*SD*_ ∝ Γ*V*_*SD*_ (Fig. 4(e)). Then, to reproduce the evolution of the stability diagram for increasing power *P*_*inc*_, we apply the average method reported above at finite bias *V*_*SD*_. In this way, we can calculate the average of the current for increasing ${V}_{AC}^{S}$ voltages. The outcome of the simulations (Fig. 4(f–h)) well reproduces the main trends of the experimental data (Fig. 4(b–d)).

To check the consistency of this phenomenological model, we compare the ${V}_{AC}^{S}$ voltages extracted from the measurements in Fig. 3 with the voltage fluctuation generated by the microwave field (*V*_*AC*_). At finite *P*_*inc*_, we calculate the root mean square voltage fluctuation as ${V}_{AC}=\sqrt{2}{V}_{AC}^{zpf}\sqrt{n+1/2}$, where *V*_*AC*_^*zpf*^ = 2.3 *μ*V is the zero point fluctuation in the center the coplanar resonator and *n* is the number of photons (Supplementary Materials). We introduce the effective coupling factor *λ* = 5 × 10^−2^ such as ${V}_{AC}^{S}=\sqrt{2}\lambda {V}_{AC}$. Finite element simulations indicate that the value of *λ* is consistent with the estimated decrease of the root mean square electric field between the electric antinode and the position of the QD (Fig. 1(b) and Supplementary Material). We also expect that the misalignment between the direction of the electric field and the NW axis contributes in determining its value.

In conclusion, we have investigated the influence of MW radiation on the transport characteristics of InAs/InP nanowire QDs and found that, above a threshold power, the MW field induces a broadening of the current peak and a current polarity reversal. These effects are relevant for microwave-assisted tunneling processes in the many-photon regime and reflect the discrete nature of QDs excited states. From these experiments we learn that a suitable choice of the working point, as well as of the electron wavefunction, enables one to control the tunneling current in strongly confined NW QD systems under external MW excitation.

## Methods

The superconducting coplanar resonator has been fabricated by optical lithography starting from commercial (Ceraco GmbH) Au(200 nm)/YBCO(330 nm)/sapphire(430 *μ*m) multilayer films. Excess Au and YBCO regions were etched by argon plasma in a reactive ion etching (RIE) chamber. In this process, gold pads were defined on top of the YBCO film in order to serve as wire bonding spots and used either to link the coplanar launchers to the external feedlines or for grounding connections. Additional leads and pads (eight per each slot) for dc measurements were fabricated by lift-off of thermally evaporated Au(100 nm)/Ti(10 nm)/sapphire films.

InAs nanowires with hexagonal section and 45 ± 5 nm diameter were grown by metal-assisted chemical beam epitaxy^31^. By changing the precursor from tert-butylarsine to tert-butylphosphine during NW growth, two 5 ± 1 nm layers of InP separated by 20 ± 1 nm of InAs were obtained. These InP layers act as tunneling barriers and define the InAs QD in the NW (Fig. 1(d)). After growth, NWs were detached from the InAs substrate by sonication in isopropyl alcohol and drop cast onto a sapphire substrate where the superconducting resonator had been previously realised. Ohmic contacts between either source (S) or drain (D) electrodes and NW were obtained by e-beam lithography followed by chemical passivation in NH_4_S_*x*_ solution, thermal evaporation of Au(100 nm)/Ti(10 nm) films and lift-off. Additionally, two local gate electrodes (G1 and G2) were fabricated in the proximity of the NW (Fig. 1(c)). The two nanogates are placed in the middle of the gap between S and D electrodes at a distance of approximately 400 nm from the NW QD. Electron transport measurements were carried out with the S electrode connected to voltage *V*_*SD*_ and the D electrode at ground potential. The latter was connected to a transconductance amplifier with gain in the 10^7^–10^9^ range for dc current measurements. No backgate bias was applied. Continuous wave microwave tones were generated by a fixed-frequency microwave source. The incident power is estimated at the input of the resonator, feedline attenuation was taken into account.

## Supplementary information

Supplementary Material
