# Supplementary material for: Microwave-Assisted Tunneling in Hard-Wall InAs/InP Nanowire Quantum Dots
Source: Sci Rep. 2019 Dec 20;9:19523. doi: 10.1038/s41598-019-56053-2 (PMC6925118; doi:10.1038/s41598-019-56053-2)
Supplement: Supplementary file 1 — Supplementary Material [file 41598_2019_56053_MOESM1_ESM.pdf]

# Supplementary Material for Microwave Assisted Tunneling in Hard-Wall InAs/InP Nanowire Quantum Dots

**Samuele Cornia[1,2], Francesco Rossella[3], Valeria Demontis[3], Valentina Zannier[3], Fabio Beltram[3], Lucia Sorba[3], Marco Affronte[1,2], and Alberto Ghirri[2,*]**

[1]Dipartimento di Scienze Fisiche Informatiche e Matematiche, Università di Modena e Reggio Emilia, via G. Campi 213/A, 41125 Modena, Italy
[2]Istituto Nanoscienze - CNR, via G. Campi 213/A, 41125 Modena, Italy
[3]NEST, Scuola Normale Superiore and Istituto Nanoscienze - CNR, Piazza San Silvestro 12, 56127 Pisa, Italy
[*]alberto.ghirri@nano.cnr.it

## Electromagnetic characterisation

Electrical characterisation and measurements under microwave radiation were performed down to 2 K in a Quantum Design PPMS by means of a low temperature probe wired with 16 dc filtered lines and 2 coaxial cables. The hybrid device was enclosed in a copper box, in which microwave and dc lines were wire bonded to a gold-plated printed-circuit board (PCB) (Fig. S1). A stage of attenuators was inserted at low temperature to suppress the heat radiated from room temperature components. The half-wavelength coplanar resonator was capacitively coupled through 140 $\mu$m wide gaps to the coplanar launchers located on the short sides of the chip that, in turns, connect the resonator to the external feedlines.

The transmission spectrum in Fig. S2(a) shows the fundamental mode of the YBCO/sapphire coplanar resonator with resonance frequency $\nu_0 = \omega_0/2\pi = 9.815$ GHz. Typical quality factor of our bare YBCO resonators are of the order of $10^4$, while the loaded quality factor, as measured for the resonator in the configuration of the hybrid device including metal contacts, is $Q_L \approx 1500$. The insertion loss is $IL = 25$ dB. To estimate the average photon population in the fundamental mode ($n$) we use the conventional formula[1]

$$n = \frac{1}{\pi h \nu_0^2} P_{inc} Q_L 10^{-IL/20}, \quad (1)$$

where $h$ is the Planck constant and $P_{inc}$ is the incident power. For the typical $P_{inc}$ values used in our experiment we estimate a photon number in the $10^6 - 10^9$ range (Fig. S2(b)).

Finite element simulations of the coplanar resonator were carried by means of a commercial software (CST Microwave Studio). Fig. S3 shows the simulated distribution of the electric field component of the fundamental mode of the resonator. From the simulation, the root mean square (rms) amplitude of the electric field at the antinode can be estimated as $E_{AC}[\text{V/m}] = 3.0 \times 10^7 \sqrt{P_{inc}[\text{W}]}$. To calculate the zero-point fluctuation, we consider an incident power corresponding to the vacuum state (-150 dBm). The rms amplitude of the vacuum fluctuation of the electric component results $E_{AC}^{zpf} \approx 3 \times 10^{-2}$ V/m.

For comparison, the root mean square value of the vacuum fluctuation on the center conductor of the resonator can be estimated as $V_{AC}^{zpf} = \sqrt{\hbar\omega_0/2C_{res}}$ (Ref.[2]). $C_{res}$ is the capacitance of the resonator, which can be calculated by analytical

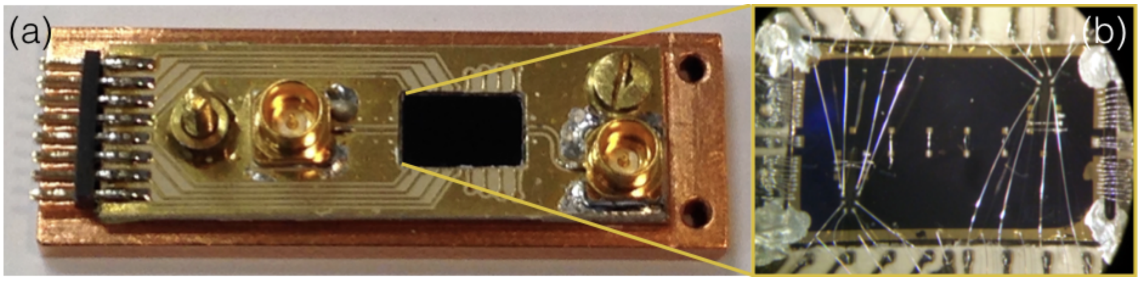


**Figure 1.** (a) Gold-plated PCB realised on a high dielectric constant AD1000 laminate. Microwave lines are connected through SMP connectors to the coaxial cables. dc lines are linked to the 16 pin connector positioned on the left side of the PCB. The board is installed into an oxygen free high conductivity copper box. (b) Device bonded on the PCB board.

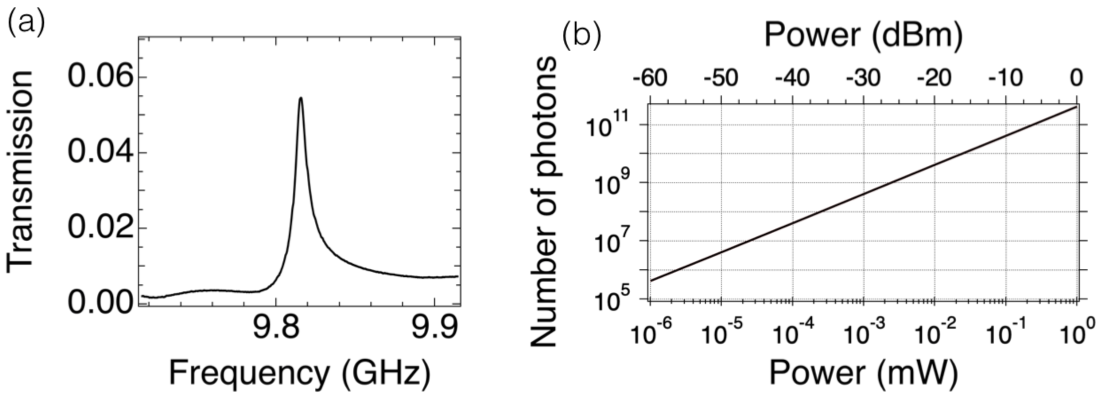


**Figure 2.** (a) Transmission spectrum of the coplanar resonator showing the fundamental mode ($T = 4$ K). The incident microwave power is $P_{inc} = -13$ dBm. (b) Average photon population as a function of $P_{inc}$.

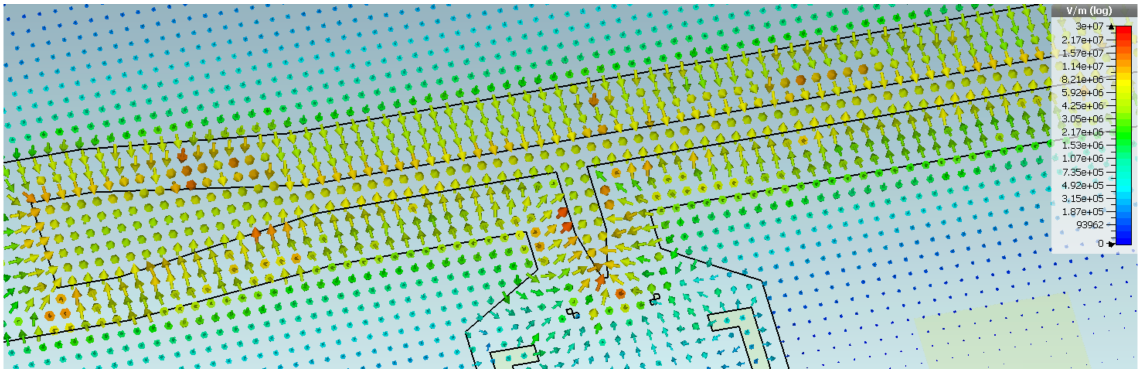


**Figure 3.** Finite element simulation of the distribution of the electric field component of the fundamental mode of the resonator. The color scale shows the magnitude of the electric field for an incident microwave power $P_{inc} = 1$ W.

techniques and in our case results 0.6 pF.[3] We thus obtain $V_{AC}^{zpf} = 2.3\ \mu$V. By considering that the distance between the center of the coplanar resonator and the ground planes is $w/2+s$, being $w = 30\ \mu$m the width of the central strip and $s = 55\ \mu$m the longitudinal separation between central strip and ground planes, we get $E_{AC}^{zpf} = 3\times10^{-2}$ V/m. This value is in excellent agreement with the outcome of finite element simulations.

From the simulated distribution of $E_{AC}$ (Fig. S3), we can estimate the rms amplitude at the position of the NW QD device as $E'_{AC}[\mathrm{V/m}] \approx 2\times10^5\sqrt{P_{inc}[\mathrm{W}]}$. This rms value is two orders of magnitude lower than that estimated at the electric antinode. For the range of incident power $-60$ dBm $< P_{inc} < -20$ dBm used in our experiments, the rms amplitude of the electric field results 10 V/m $< E'_{AC} <$ 100 V/m. We note that the effective length calculated from the ratio $V_{AC}^{lead}/E'_{AC} \sim 30\ \mu$m suggests that the electromagnetic wave effectively couples to the leads to modulate their voltage.

## Tunneling rate

The tunneling rate of the QD at $V'_G = 0.86$ V was determined by fitting the Coulomb peak measured in the low bias limit with the equation[4]

$$G = \frac{I_{SD}}{V_{SD}} = \frac{e^2\Gamma'}{k_B T'} \frac{1}{4\cosh^2\left(\frac{e\alpha(V'_G - V_G)}{2k_B T'}\right)}, \tag{2}$$

where $\alpha$ is the lever arm of the gates whilst $e$, $h$ and $k_B$ are respectively the elementary charge, the Planck and the Boltzmann constants. From the experimental data (Fig. S4) we extracted $\Gamma' = 1.0\pm0.1$ GHz and $T' = 2.5\pm0.1$ K. The latter is compatible with the measured temperature of the experiment ($T = 2$ K).

For Coulomb peaks at $V_G > 1$ V the tunneling rate was simply estimated by the maximum value of the current at fixed bias.

## Additional measurements

Fig. S5 shows the evolution of the Coulomb peak at $V'_G$ for increasing values of $P_{inc}$ (Fig. S2). In this case the frequency of the microwave tone is $\omega_1/2\pi = 9.810$ GHz. $I_{SD}(V_{SD})$ characteristics measured for increasing power $P_{inc}$ show features comparable to those reported in the main article for $\omega_0/2\pi = 9.815$ GHz. The vertical shift of the measured curves in terms of incident microwave power follows as a consequence of the reduced transmission at $\omega_1$. No significant frequency dependence was observed within the resonator bandwidth.

Fig. S6(a) shows the stability diagram measured near $V'_G = 0.86$ V at the temperature $T = 8$ K. Respect to the experimental data taken at $T = 2$ K (main article), the Coulomb peaks are broader due to the higher temperature. Current polarity reversal and peak broadening are visible in the presence of the microwave drive (Fig. S6(b)).

## References


1. Sage, J. M., Bolkhovsky, V., Oliver, W. D., Turek, B. & Welander, P. B. Study of loss in superconducting coplanar waveguide resonators. *J. Appl. Phys.* **109**, 063915, DOI: 10.1063/1.3552890 (2011). https://doi.org/10.1063/1.3552890.
2. Wallraff, A. *et al.* Strong coupling of a single photon to a superconducting qubit using circuit quantum electrodynamics. *Nature* **431**, 162–167, DOI: 10.1038/nature02851 (2004).
3. Lancaster, M. J. *Passive Microwave Device Applications of High-Temperature Superconductors* (Cambridge University Press, 1997).
4. Van Houten, H., Beenakker, C. W. J. & Staring, A. A. M. *Coulomb-Blockade Oscillations in Semiconductor Nanostructures*, 167–216 (Springer US, Boston, MA, 1992).

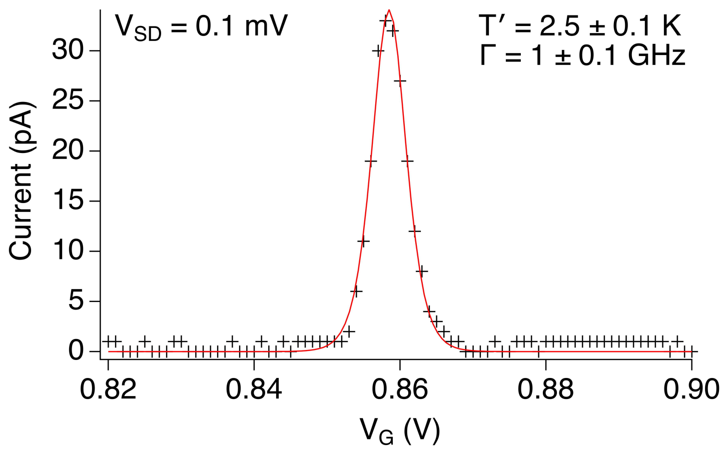


**Figure 4.** Current $I_{SD}$ measured as a function of the gate voltage $V_G$ at $V_{SD} = 0.1$ meV (symbols). The fit with Eq. 2 is displayed by the solid line.

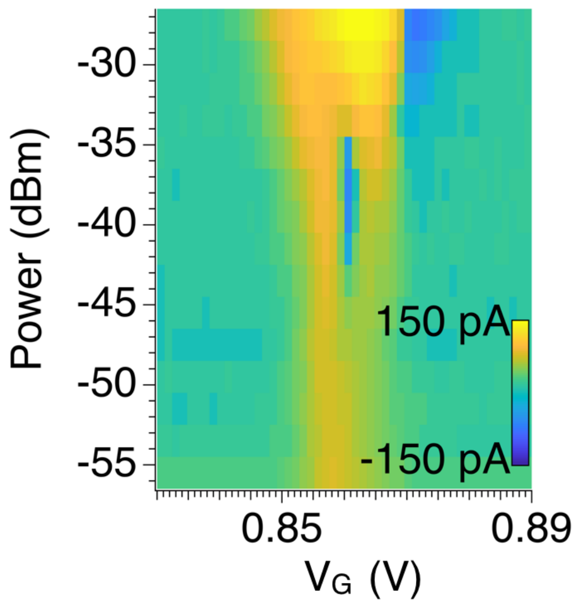


**Figure 5.** Evolution of the $I_{SD}(V_G)$ characteristics in the presence of a microwave tone of increasing power $P_{inc}$ and frequency $\omega_1/2\pi = 9.810$ GHz. The temperature is $T = 2.4$ K.

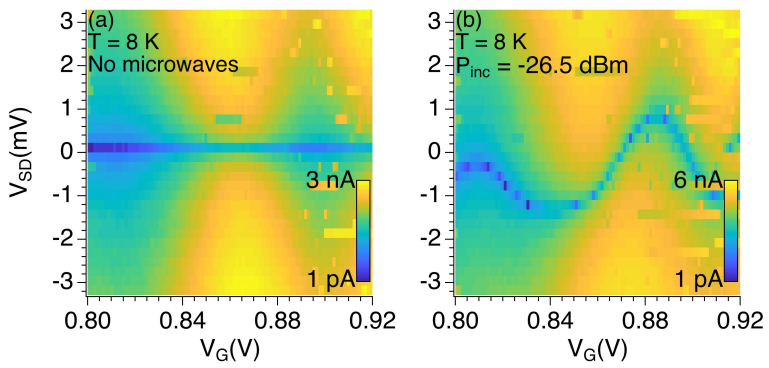


**Figure 6.** Current ($I_{SD}$) maps taken at the temperature $T = 8$ K. (a) $P_{inc} = 0$, (b) $P_{inc} = -26.5$ dBm and $\omega = \omega_0$.
